# GeoDanceHive: An Operational Hive for Honeybees Dances Recording

**DOI:** 10.3390/ani13071182

**Published:** 2023-03-28

**Authors:** Sylvain Galopin, Guillaume Touya, Pierrick Aupinel, Freddie-Jeanne Richard

**Affiliations:** 1 AgroParisTech, 22 place de l’Agronomie, F-91220 Palaiseau, France; 2 LASTIG, University Gustave Eiffel, IGN-ENSG, F-77420 Champs-sur-Marne, France; 3 Laboratoire Écologie et Biologie des Interactions—UMR CNRS 7267, Université de Poitiers, F-86000 Poitiers, France; freddie.jeanne.richard@univ-poitiers.fr; 4 Institut National de Recherche Pour l’agriculture, l’alimentation et l’environnement, Le Magneraud, UE APIS 1255, CS40052, F-17700 Surgeres, France

**Keywords:** honeybee, waggle dance, animal communication, foraging, automation, behaviour

## Abstract

**Simple Summary:**

Honeybees are known for their ability to communicate about resources in their environment. They inform the other foragers by performing specific dance sequences according to the spatial characteristics of the resource. The purpose of our study is to provide a new tool for honeybees dances recording, usable in the field, in a practical and fully automated way, without condemning the harvest of honey. We designed and equipped an outdoor prototype hive, later called “GeoDanceHive”, allowing the continuous recording of honeybees’ dances and their analysis. The use of the GeoDanceHive is designed for a wide range of users, who can meet different objectives, such as researchers or professional beekeepers. Thus, our hive is a powerful tool for honeybees studies in the field and could highly contribute to facilitating new research approaches and better understanding landscape ecology of key pollinators.

**Abstract:**

Honeybees are known for their ability to communicate about resources in their environment. They inform the other foragers by performing specific dance sequences according to the spatial characteristics of the resource. The purpose of our study is to provide a new tool for honeybees dances recording, usable in the field, in a practical and fully automated way, without condemning the harvest of honey. We designed and equipped an outdoor prototype of a production hive, later called “GeoDanceHive”, allowing the continuous recording of honeybees’ behavior such as dances and their analysis. The GeoDanceHive is divided into two sections, one for the colony and the other serving as a recording studio. The time record of dances can be set up from minutes to several months. To validate the encoding and sampling quality, we used an artificial feeder and visual decoding to generate maps with the vector endpoints deduced from the dance information. The use of the GeoDanceHive is designed for a wide range of users, who can meet different objectives, such as researchers or professional beekeepers. Thus, our hive is a powerful tool for honeybees studies in the field and could highly contribute to facilitating new research approaches and a better understanding landscape ecology of key pollinators.

## 1. Introduction

Only humans and honeybees can communicate factual information about distant localities [1]. The Apis family includes several species of bee (*Apis mellifera*, *Apis indica*, *Apis dorsata*, *Apis florea*) known for their ability to communicate with each other [2]. To do this, the honeybees use a very elaborate system of dances called waggle dances. The European honeybee (*Apis mellifera*) has the most advanced dance system, allowing it to transpose the position of the sun in the darkness of the hive [2]. Back in the colony, they can communicate on the approximate location of resources to recruit other nestmates. The characteristics of the dance indicate the direction, the distance and the profitability of a search area [3,4]. The approximate direction is given by the angle formed between the trajectory of the honeybee during its waggling and the vertical line of the hive. This angle represents the angle between the sun, the hive and the search area. The approximate distance is given by the duration of the honeybee waggling [5,6,7,8]. Although the accuracy of this dance was sometimes disputed [9,10,11,12], other studies confirm its effectiveness and improved the knowledge of the characteristics of the dances and their meaning [6,13,14,15]. The accuracy of nestmate recruitment was also later confirmed using harmonic radar [16] and recently described by a new Three-Phase Model (send, search and attract) [1,17]. Herta Knaffl, part of von Frisch’s research group, was the first to use the waggle dances to study the foraging areas of a colony [1]. Monitoring and mapping the evolution of foraging areas are fundamental to understaning pollinators at the landscape scale, and to better identify the floral origin of hive products.

Observation hives existed long before the invention of the first flat glass in France in the 1600s, thus underlining the interest that men have always had in studying honeybees [18]. The first hive containing a small side glass, later called “side-glass”, was described in 1655 [18,19]. In 1851, L.L. Langstroth made the first practical movable-frame tiered hive including the correct comb-space and bee-space above, below and around frames [18]. In 1857, Langstroth made the contemporary “side-glass” observation hive using its full-sized frame hives with two or more glass walls. The same year, he made the demonstration of a “single-frame” observation hive, being a “single-comb” hive with a movable frame, coming from one of his hives resolving the problem of the comb building [18]. In 1866, T.W. Woodbury proposed the first observation hive with several stacked frames, later called “array-frame” [18]. In 1869, C. Dadant described a new movable-frame hive [18] with the brood box being deeper than in the Langstroth hive. Dadant hive was designed to provide more natural conditions by increasing the egglaying space (space available for the queen) in the hive body and to reduce hive inspection time [18,20]. Moreover, the volume of the Dadant (54 L), being greater than the Langstroth (44 L), allows to increase the reserves of honey necessary for overwintering [20,21]. Nowadays, in Europe and worldwide, the Dadant hive is the most widely used [21,22]. In 1889, J. Hoffman designed frame spacers to maintain the correct comb space [18]. In the 1920s, K. von Frisch used an “array-frame” with two vertical frames to observe the honeybees’ dances [18]. Today, for more than a century and a half we still continue to use the same observation hives invented in the 1860s with either the “side-glass” or “array-frame” shapes. With new technologies, we proposed here a new type of observation hive which is an improvement of the “side-glass”. This new type, later called “inner-glass” is characterized by a glass positioned on one side of the brood frames and in front of cameras, all being inside the hive body.

**Figure 1:**
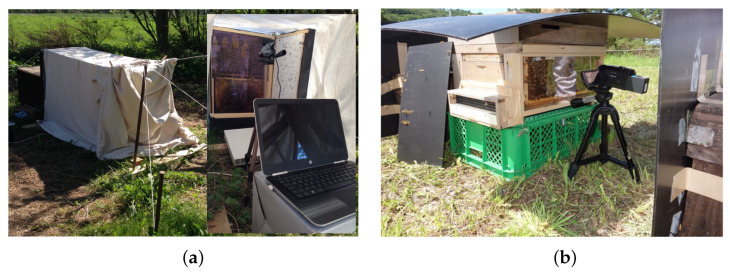
Example of outdoor studies with observation hives: (**a**) Observation hive of type “array-frame” with two vertical frames and sheets as a sun visor [26]; (**b**) Observation hive of type “side-glass” with 10-frame Langstroth hive and black plastic panels as sunlight shield [27].

Automatic analysis of honeybee dances is divided into two main steps. The first step is the automation of recording and the second is the automation of decoding. Here, in the current article, we address the first step. Several indoor hives dedicated to the recording of the dances have been prototyped but are inherently unusable in the field [23,24,25]. There are currently no turnkey solutions dedicated to recording dances in the field. Current solutions [26,27] are difficult to deploy, in particular, because of reflections issues on the glasses, which are difficult to manage (Figure 1) and due to the lack of integration between the components of the system (hive, camera, computer…).

Monitoring honeybees foraging throughout the season requires systematic recording of honeybees dances. Thus, it’s necessary to design a device allowing a permanent recording in any weather conditions operating autonomously without disturbing the honeybees. The recording equipment must be practical, reliable and easy to install to facilitate observation in various environments. To reach this objective, we designed a fully automated hive dedicated to recording the honeybee dances. Our recording hive has many technical advantages such as resistance to various types of weather and sunlight protection. Indeed, misdirections can occur when honeybees can see the sky [1]. Its compact dimensions compliance with beekeeping standards and the all-in-one design of its integrated recording system, make it easily transportable and reusable in any apiary.

## 2. Materials and Methods

A custom “inner-glass” observation hive, called GeoDanceHive, was designed with the most common and widely used hive standard, namely, the Dadant format, to facilitate its deployment [21,22] but the underlying concept can be easily adapted to the Langstroth format and many others. Nowadays, unfortunately, there is no strict standard regarding the dimension of the Dadant hive, each country has its own standard and each manufacturer can make its own design. The exact dimensions of the GeoDanceHive are given in Appendix A.

GeoDanceHive was designed to integrate an embedded system equipped with two cameras mounted with low distortion lenses and controlled by a microcomputer also managing infrared lighting. All the electronics are assembled in a specially designed box for easy handling and mounting on a Dadant Hoffman frame.

The Magneraud site in France was chosen to test the GeoDanceHive in the field throughout the season due to the presence of an experimental team specialized in honeybees from the French National Research Institute for Agriculture, Food and the Environment (INRAE), which can carry out field experiments with feeders to test our prototype.

For the current study, we used the common European dark bee subspecies (*Apis mellifera mellifera*) characterized by their capacity to dance inside the hive.

### 2.1. GeoDanceHive

**Figure 2:**
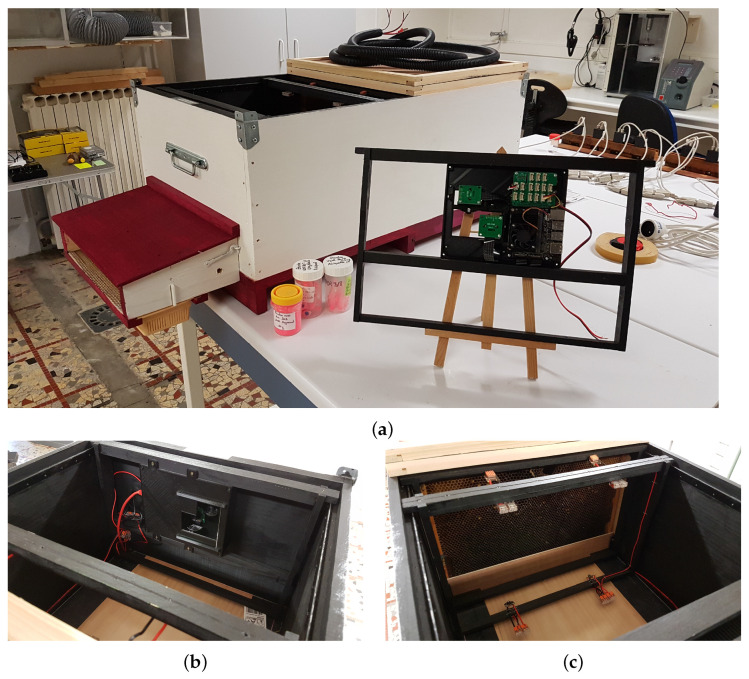
Photos of the hive before implantation in the apiary: (**a**) Full overview; (**b**) View of the embedded system in place; (**c**) View of the dance floor.

**Figure 3:**
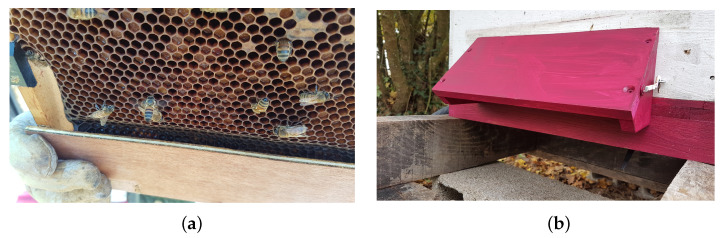
(**a**) Wood ramp with gnawed wax; (**b**) Awning for use when pollen trap is removed.

The GeoDanceHive (Figure 2) comprises two compartments, each corresponding to a 10-frame Dadant hive. Its dimensions are the same as two regular hives merged on the long side, this compact size facilitates transport and allows you to put two GeoDanceHives in opposition on a pallet. The first part, later called the nest, contains the 10 frames of the swarm and can be managed like a standard hive. The second part later called studio is a black paint wooden box used as a recording studio holding the embedded system equipped with two cameras (Figure 2b) and a frame of IR lights (Figure 2c). The two compartments are separated by an “invisible” glass mounted on a custom divider to maintain the colony temperature over the seasons (Figure 2c). The bee-space between the glass and the side frame is 5.5 mm to prevent honeybees from climbing on each other. At the top, the bee-space is ensured by the Hoffman frame self-spacing and at the bottom by Hoffman frame plastic adaptors mounted on the frame end bars (Figure 3a). Both hive’s length sides are equipped with folded smooth strips to hold the Dadant Hoffman frames used to maintain the correct comb-space (Figure 2b,c).

The entrance of the hive is located on the long side of the studio (Figure 2a and Figure 3b) and runs parallel to the frames. The honeybees are channelled into a 9mm bee-space between two pieces of wood until the first frame (Figure 2c). These pieces of wood are covered with plexiglass until the “invisible” glass to keep the studio part free of honeybees (Figure 2b,c).

**Figure 4:**
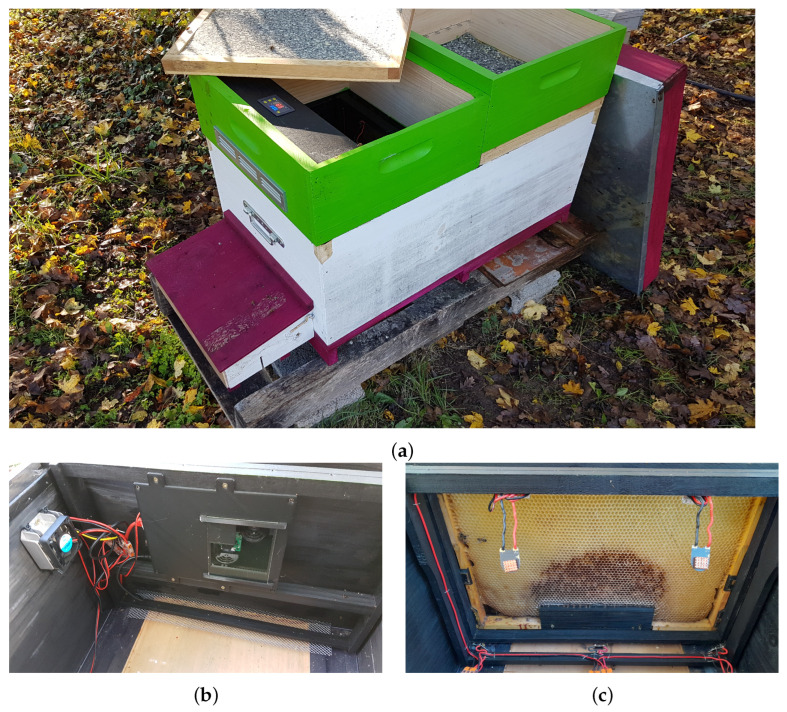
Photos of the hive at the end of the season: (**a**) Ventilated super with empty super to compensate for height difference; (**b**) View of the embedded system in place with the ventilated regulator and the lower mesh; (**c**) View of the dance floor frame with the new shorter ramp and the five LEDs adjusted on the frame to maximize the lighting while avoiding reflections.

**Figure 5:**
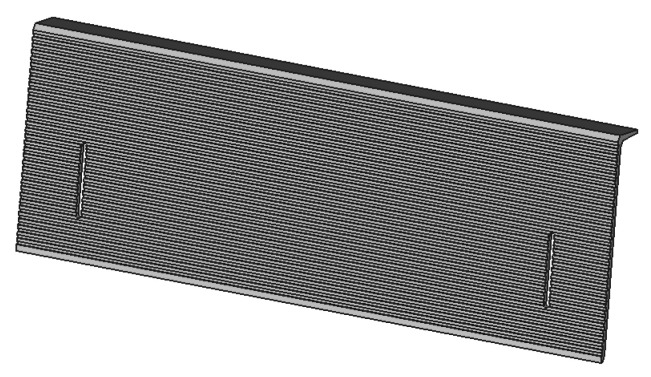
Small ramp used to canalize the honeybee on the first frame.

A ramp has been added on the first frame to facilitate the ascent of the honeybees on the Figure 2c). The first frame must not have capped cells on its lower part to maximize the vibrations transmitted by the dances and therefore the recruitment [1]. Initially, the ramp was in wood and had the inner hive width to guide the honeybees to go upwards. As the honeybees gnawed the frame’s wax to make a shorter path (Figure 3a), the current ramp (Figure 4c and Figure 5) is now shorter than the frame to allow non-dancing honeybees to pass directly under the first frame. The ramp must be mounted on a frame whose bottom bar is less wide than the end bars. A space of 2 mm (thickness of the ramp) is necessary to fix the ramp without reducing the bee-space between the ramp and the glass which must remain at 5.5 mm.

An awning has been added over the hive entry to reduce the brightness at the entrance (Figure 3b). It can easily be replaced by a pollen trap to collect pollen samples (Figure 2a) but a small piece of wood must be added to the front of the trap to reduce the brightness. Both the awning and pollen trap can be removed to facilitate the displacement of the GeoDanceHive.

To avoid overheating and system shutdown during the summer heat, a customized ventilated super has been designed (Figure 4a) to be placed on the top of the studio. It was equipped with 5 WINSINN brushless high-speed fans that can run up to 16 h non-stop (FAN50105VH), a Steinberg14 galvanized steel ventilation grille with mosquito net (300 × 50 mm), a digital temperature controller (KUNSE W1018 5V) and a 5 V power supply (17679 PSU-5V4A-5.5-2.1-EU by Waveshare). The difference in height with the brood box was compensated by a super placed on it. The nest’s inner cover was positioned above or below depending on the desired volume (i.e., depending on the wish to harvest honey or not). A rectangular hole into the plexiglass was made under the frame of the embedded system and covered with a mesh (folded one centimeter along the length to stiffen it) to allow the suction of outside air by the depression caused by the fans (Figure 4b).

### 2.2. Embedded System

#### 2.2.1. Optical System

The mean frequency of the honeybee waggle dance is approximately 13 Hz and can increase to more than 18 Hz (μ = 12.67 Hz σ = 1.89) [28]. To respect the Nyquist–Shannon sampling theorem, we decided to use an acquisition frequency of 60 Hz and to record the whole frame (41 × 26 cm). The recording is done by two ArduCam NOIR (No InfraRed) cameras based on the Sony 8MP IMX219 sensor (B0152 by ArduCam), fully compatible with Raspberry Pi Camera V2.1 and with an M12 lens mount. The sensor pixel size is 3280 × 2464 but to reach 60 frames per second, the video resolution is reduced to 720p after pixels binning and cropping.

Infrared light at 850 nanometers (nm) of wavelength was chosen to avoid honeybee disturbance because honeybees have a spectral color vision between 300 and 650 nm [29,30]. Red colors produce a higher contrast than other colors (green and blue) composing a classic RGB image and reflecting more the predominant warm colors (yellow and orange) of the honeybee body [31]. Thus, five 3 W high-power 850 nm infrared LEDs (10670 by Waveshare) were used to illuminate the dance floor. The LEDs were fixed on a Dadant frame to keep the possibility of adjusting the distance to the glass. Each LED was equipped with a copper Self Adhesive Heat Sink (7133 by Waveshare) to dissipate the heat that they produced. The lighting is being managed by the software, and the photoresistors (ambient light detectors) have been removed. To improve light diffusion, the lenses of the LEDs have been removed too.

Two special Arducam low-distortion lenses have been used in order to have two orthorectified images without post-processing and with a recoil of 40 cm. The first lens is used for the detection of the dancing honeybees and is characterized by a horizontal field of view (hfov) of 75° on ¼” Raspberry Pi (RPI) camera chipset with a distortion inferior to 1.5% (M27280M07S by Arducam). The second lens is used to evaluate the honeybee motion inside the new hive and is characterized by an hfov of 90° on the same camera and a distortion inferior to 1% (M40210M09S by Arducam).

Regarding the “invisible” glass (Artglass Extra Blanc AR70 by Groglass) used to separate the studio from the nest, the reflection is inferior to 1%. Despite the low reflectance of the glass, the spotlights must be well-adjusted to prevent their reflections from being on the dance floor.

#### 2.2.2. Electronic

**Figure 6:**
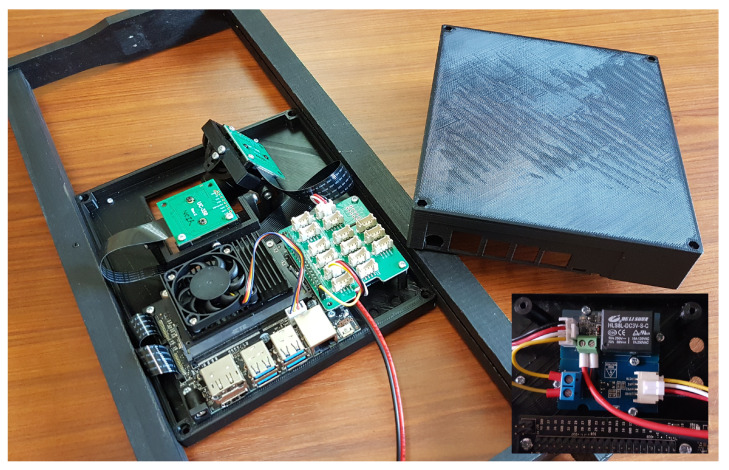
The embedded system with the relays placed under the Grove hat and the two antenna holes in the lower right.

The microcontroller unit (MCU) used was the Jetson Nano Developer Kit 4GB—Version B01 (Figure 6). The system was powered by the 4 A input to get the maximum capacity of the MCU. The default Jetson dissipator was replaced by a smaller GeeekPi dissipator equipped with a pulse width modulation (PWM) fan. The RJ45 port was used to connect the system to the internet network. Both camera serial interfaces (CSI) were linked to the cameras via two 20 cm 15-pin flexible flat cables (FPC). A Grove base hat for RPI (Grove 103030275 by Seeedstudio) was plugged into the Jetson general purpose input/output (GPIO) 40-pin port. The Grove System was selected for the richness of its modules and the possibility to add additional sensors to the system (light, temperature, etc.). We also used an optocoupler relay M281 (Grove 101020603 by Seeedstudio) to switch a mechanical relay (Grove 103020005 by Seeedstudio) to supply the current of 3 A required by the 5 IR LEDs (3 W/5 V).

#### 2.2.3. Power Supply and Network

A ventilated empty independent hive, called “power supply hive” (PSH), contained the electrical and data networks. In this hive, an ethernet switch broadcasted the network via ethernet cables and a 230 V/12 V ventilated transformer was supplying the power to the embedded systems. This last one was associated with a ventilated regulator (8–40 v/5 v) (BgoodVision 10 A 50 W) supplying the LEDs, the Jetsons and the fans (Figure 4b) inside the studio (Figure 4b). This regulator is necessary to absorb voltage variation induced by temperature differences. Before using this set up the hive was powered by three meters wires connected to a 230 v/5 vs. transformer lodged into the PSH. However, with high temperatures, the resistance of the cables increased leading to voltage drops on the Jetson and conducted to the shutdown of the system. Moreover, this 12 vs. tension offers the possibility to connect the hive to a solar power station, leading to an energy-independent system. We also used low DC voltages to limit electromagnetic pollution [32,33]. Cables with a section of 2 × 0.75 mm^2^ supplied all the electronic elements and the external cables (electrical and ethernet) were protected by a sheath.

#### 2.2.4. 3D Design

**Figure 7:**
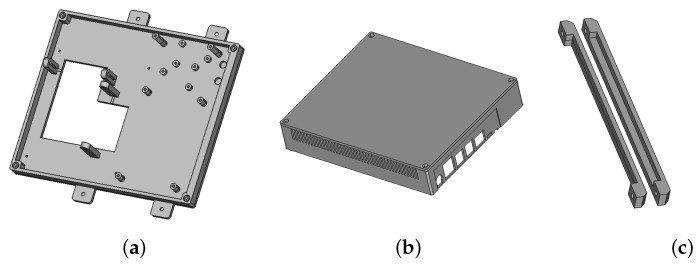
(**a**) Front of the box; (**b**) Box cover; (**c**) Glass holders.

In order to fix all the electronic elements together, with the possibility of adjusting the alignment of the camera, it was necessary to create six different parts. The first part was the front of the box (Figure 7a). It contained an “L” shaped hole and four legs to hold the two cameras. Six studs of 3mm were designed to support the Grove relays under the Grove hat which was supported by two studs of 22 mm and the Jetson GPIO. The Jetson was fixed on four studs of 9mm. Four ears were present to fix the box on the wood frame. Two of them were aligned with the detection camera to facilitate the centering of the box on the frame. The second part was the box cover (Figure 7b). On one side, cavities have been added to allow access to the Jetson ports. Another niche was added to leave a pass to the LED supply wire managed with the relays. Two ventilation grilles with 2 mm wide holes have been made at the bottom and top of the cover to facilitate natural air convection. The third and fourth parts were two glass holders screwed on the box front at the top and the bottom of the camera hole (Figure 7c). A small “invisible” glass (84 × 108 × 2 mm) (Figure 2b) keeps the electronics free of honeybees and protects the camera lens. The holding system was modeled in order to easily change the glass without tools.

**Figure 8:**
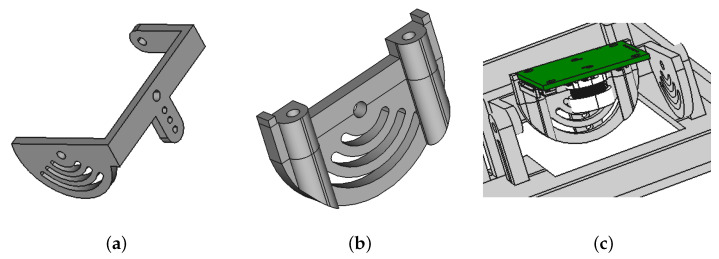
(**a**) First camera mount; (**b**) Second camera mount; (**c**) Camera mount.

The fifth and sixth parts (Figure 8) were two camera mounts with the ability to adjust roll and pitch.

Finally, the last part is a small ramp (Figure 5) used to canalize the honeybees on the first frame. Two oblong holes allow the fixation on the bottom bar of the frame. A small cap at the top of the ramp was added to prevent wax gnawing.

All the different pieces have been modeled on FreeCAD and printed on an Artillery Sidewinder-X1 3D printer using Ultimaker Cura. The time to print all pieces was 29 h and 39 min (Cover 14.95 h, Front 10 h, Ramp 3.15 h, First mount 0.65 h, Glass holders 0.55 h, Second mount 0.35 h).

### 2.3. Embedded Computer Software

**Figure 9:**
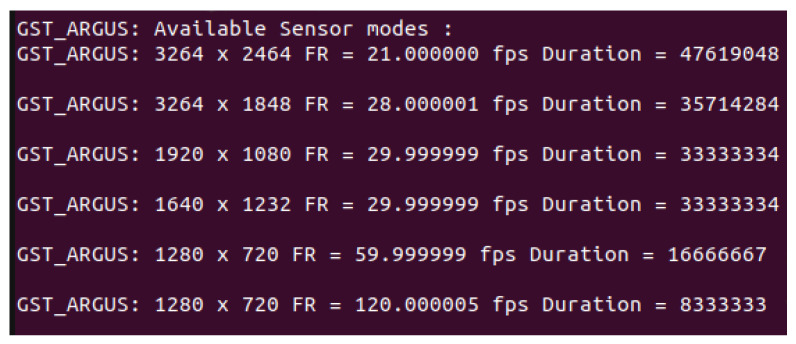
IMX219 camera sensor modes.

The Jetson was set up with a Jetson nano Jetpack 4.6.1 sd card image (L4T 32.7.1). Python scripts were coded to pilot the cameras and the LEDs. The main script is using two GStreamer pipelines (chaining of capture, encoding, wrapping and writing) taking advantage of the Nvidia hardware acceleration to record simultaneously two types of video in MPEG-4 container format. The first pipeline uses the fifth sensor mode (Figure 9) of the IMX219 camera sensor and an h264 codec (high profile, 3.2 level) to generate the detection videos at a resolution of 720p and a framerate of 60 fps. The 120 fps of the sixth sensor mode makes it possible to improve acquisition by capturing at 120 Hz. However, the exposure time in this mode will be reduced by half for equal resolution which will alter the precision of dance visual analysis. The second pipeline uses the fourth sensor mode and an h264 codec (high profile, 4 levels) to generate the hive videos at a resolution of 1640 × 1232 and a framerate of 30 fps.

During recording, the LED states are managed by the script. By default, the duration of the videos was set to 10 min (to limit the video weight) with the possibility to specify gaps between the records (set to 0 by default). Capture times (start and end times) were scheduled with a scheduled task. Two scripts are used to preview (via an ssh connection) the videos inside the hive without recording to adjust the alignment of the camera. A third script gives the possibility to switch the light. The last script wrapped in a scheduled task is used to synchronize the videos with a server, one minute after the end of the recording. During the night, the videos are saved into a dedicated network area storage of 12TB (NAS). The code was versioned on a free and open-source distributed version control system (GIT) and freely available on a public GIT repository.

### 2.4. GeoDanceHive Field Assessment

**Figure 10:**
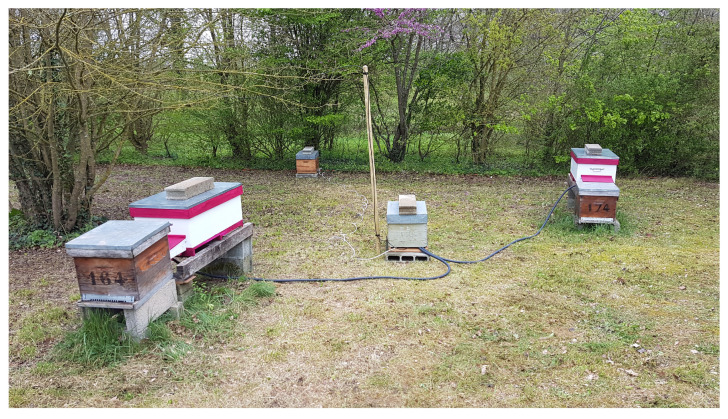
Installation of the two prototypes in the apiary with the old hives in front waiting for good weather for the transfer and the “power supply hive” (PSH) in the middle.

**Figure 11:**
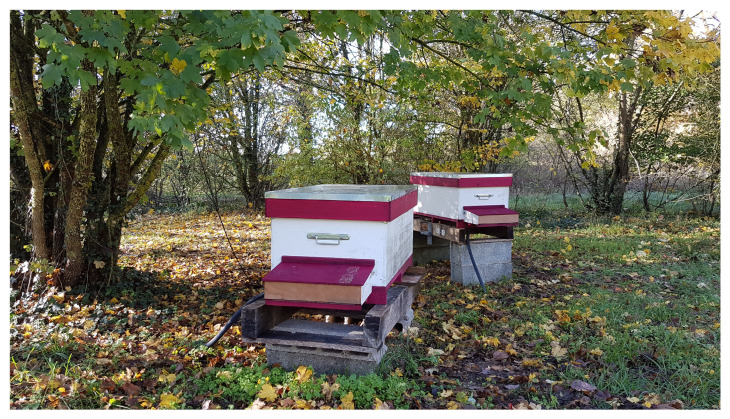
The prototypes in the apiary at the end of the season, moved under a tree to take advantage of its shade and with a small piece of wood added on the front of the pollen trap to reduce the brightness.

The GeoDanceHive was tested for all the beekeeping season by an INRAE professional beekeeper (Figure 10 and Figure 11), which was in charge to maintain the honeybee colonies healthy and strongly populated.

In order to test the GeoDanceHive and to provide an example of the output, we used a feeder with unscented syrup to limit plundering by other colonies in the apiary. To compare our results with other similar studies [28,34], the feeder was placed in a wood at about 230 m from the hives on a sunny day. Several hundred foragers were collected from the GeoDanceHive and released inside the feeder. The capture-realized method was repeated until a consistent number of dances scoring the feeder position was clearly identifiable on the videos.

To facilitate the analysis, a Python script was developed to add a static grid to the videos. An observer collected and did a visual analysis of video dances frame by frame and grid cell by grid cell. For each waggle phase (center line of the dance’s eight-figure), the angle of the phase was deducted from the angle from the vertical and the mean path orientation of the phase, and the duration of the phase from the difference between the start and the end frames times. The data were directly filled into a dedicated Postgresql 14.6 database. After verification and correction, the data was exported from the database by a Python 3.10.6 script to generate a map of all the vector endpoints deduced from the waggle phase information and a heat map produced with the dance information. The maps were produced with Inkscape 1.2 and QGIS Desktop 3.28.

## 3. Results

### 3.1. Videos Resolutions and Volumes

**Figure 12:**
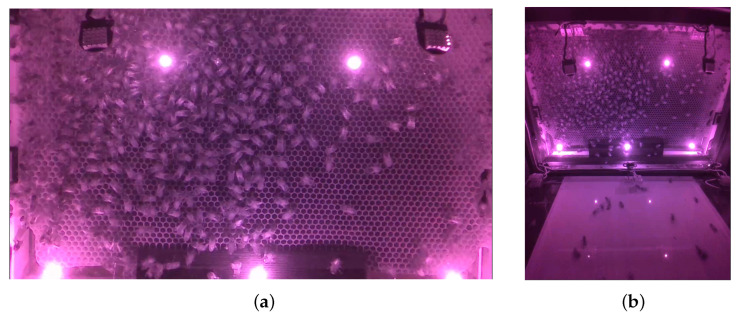
Example of view from the cameras: (**a**) View from the first camera; (**b**) View from the second camera.

The recording was made over a 6 months period from 11 April 2022 to 20 October 2022. In April it was launched manually on the desired day to test and improve the system. After that, the acquisition was automated, firstly for a recording every two days and every day from June to October when the video volume was confirmed and the NAS available. With the two prototype hives, and so the 4 cameras, we recorded more than 2000 h of acquisition by the camera over six months and about 10 TB of data. The frontal camera gives orthorectified imagery without numeric treatment of all the wax included in a Dadant frame (41 × 26 cm) (Figure 12). The generated 16/9 h264 MPEG-4 video has a resolution of 720p (1280 × 720 px) at 60 fps. Taking as reference the frame’s inner width which is matching well the video width, the spatial resolution is about 1.76 px/mm and a 15 mm high honeybee will be 26 px on the screen. The h264 codec is a dynamic codec, the video weight depends on the motion and on the number of honeybees moving on the frame. The average video size for the season is 200 Mo with a maximum of 260 Mo.

The second camera used to evaluate the honeybee motion inside the new hive provides wider imagery (Figure 12). The generated h264 MPEG-4 video has a resolution of 1640 × 1232 px at 30 fps. The imagery being inclined in relation to the frame and the distance to the captured plan varying, the spatial resolution of the video depends on the point of interest. The average video size for the season is of 210Mo with a maximum of 280Mo. The videos were used to confirm the tunnel effect between the entrance of the hive and the first frame and to check the lack of dances at this level. This confirms that honeybees prefer to dance on the combs to attract followers from a distance [1].

### 3.2. Encoding and Sampling Quality

Videos recorded by GeoDanceHive were analyzed by visual observation (by a human observer). For example, the video of 21 June 2022, recorded on a feeder day between 12:49 and 12:59 p.m. contains 1491 waggle phases and 141 dances, i.e., 14 dances/min. This large number of dances seems to show that the repositioning of the hive entrance perpendicular to the first frame is successful and that the location of the dance floor in the hive can be influenced by the morphology of the hive.

The video quality also allows the analysis of dances by human eyes, which is a necessary step to validate the encoding parameters, in particular for its good weight/quality compromise. The hive makes it possible to quickly capture large samples of data (waggle phases and dances) and to produce maps.

**Figure 13:**
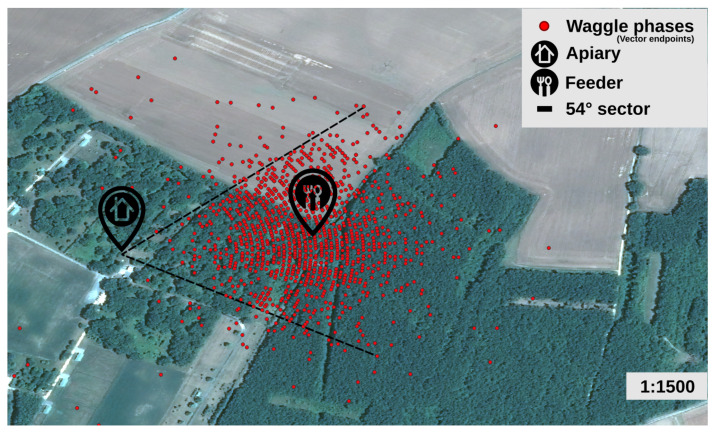
Map of the vector endpoints deduced from 1491 waggle phases decoded by a human observer on 10 min video (21 June 2022) with Pleiade image background (11 June 2022). The position of the feeder is about 230 m east of the GeoDanceHive.

**Figure 14:**
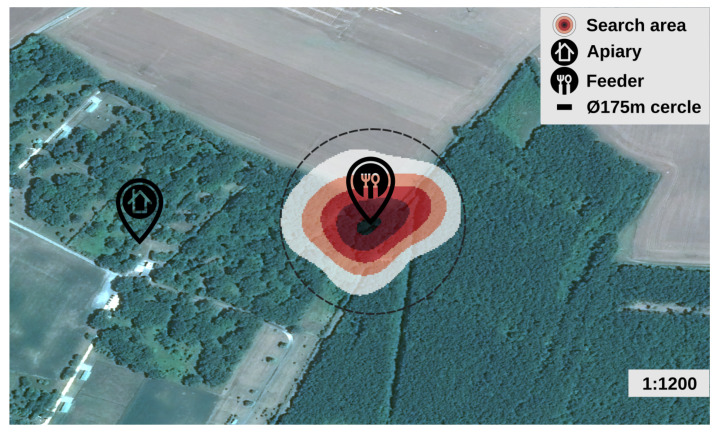
Heat map of the search area deduced from 141 dance vector endpoints decoded by a human observer on 10 min video (21 June 2022) with Pleiade image background (11 June 2022). The position of the feeder is about 230 m east of the GeoDanceHive.

The majority of the points on the waggle phases map (Figure 13) are centered on the feeder and included in a visual sector of 54° which is in agreement with the known inaccuracy of waggle phase direction [1,35]. A large number of points allowed us to see the concentric curves due to the sampling frequency (60 Hz). The approximate distance between the two curves is 6 m. Sampling quality is sufficient in relation to the precision of the search area visible on the map (Figure 14).

The search area map was produced by applying a heat map style (30 mm radius and 0, 20, 40, 60, 80, 98, 100% color palette) on the vector endpoints deduced from the dances. The search area is centered on the feeder and included in a circle of 175 m in diameter. The determined distance calibration factor was about 380 m/s (dance time: η = 0.60 s μ = 0.63 σ = 0.14 for dances included in the 54° sector). For a 13 Hz waggle phase frequency, the waggle phase accuracy is 29 m per waggle movement. This value is close to the results obtained in other studies [1]. Sampling quality at 6 m is sufficient compared to the waggle phase accuracy.

### 3.3. Impact on Beekeeping Practices

The beekeeper in charge of the hives did not report any problems or any discomfort during the management of the colonies populating the GeoDanceHive. All the materials were compatible. To avoid system shutdown, all parts have been ventilated and the hives were placed in the shade of a tree. The system was resilient over the summer heat (up to 42 °C in the shade in the study apiary that season) and over the full entire season.

## 4. Discussion

As part of this work, we have produced a new device equipped with two cameras to study and decode the waggle dances of honeybees in the field. We tested this device during the 6 months of a beekeeping season in France and were able to demonstrate the lack of impact for professional beekeepers. The first camera of the system is dedicated to the analysis of the dances and the second to evaluate the honeybee motion and can also be used a posteriori to count the entries and the exits. The field assessments confirmed the hive design and the quality of encoding and sampling.

Compared to the existing outdoor prototype hives [26,27], the GeoDanceHive contained many novelties and improvements. First, our prototype can be quickly deployed in any apiary with no need for shelters (to protect the equipment from the weather or from the sunshine that could be reflected on the glass surfaces and disturb the honeybees). Secondly, unlike “array-frame” observation hives, traditionally used for the study of dances, the GeoDanceHive is made with standard beekeeping equipment. Thus, the management of the hive is the same as for any hive, which greatly facilitates handling by beekeepers and harvesting of honey. GeoDanceHive size allows putting two per pallet making it compatible with the use of pallet truck. Thirdly, with the camera being fixed on a frame, the optical adjustments are greatly simplified. Finally, the system power supply being 12v, it is possible to install the GeoDanceHive both on the roof of a building and in an isolated apiary with a solar power plant.

Shooting schedule times are easily configurable and can also be supplemented with external sensors (light, temperature, etc.) as needed by plugging them into the Grove ports left available. The glass and the built-in infrared lighting allow the honeybees to be observed at any time and so to record without interruption an enormous amount of data. Unlike current systems, the device has an ethernet port to send the data in real time. It can easily be equipped with a WIFI/BlueTooth card and pre-holes have been provided in the box to fix the two antennas supplied with the optional card (Figure 6). In the event of a network failure, local memory space can be added by plugin USB keys in the four USB ports left available, allowing an operator to come and retrieve the data directly on-site.

The light, framing and resolution being fixed and constant, the videos acquired via the first camera can be processed by computer vision software in order to automate the detection of dances. In the same way, the use of artificial intelligence for detection also becomes possible with the acquisition of a large number of videos which allows for constituting the datasets necessary for the learning process.

## 5. Conclusions

In conformity with the objectives, a fully automated hive, GeodanceHive, has been conceptualized and tested on the field over the beekeeping season to facilitate the honeybee waggle dance monitoring. The prototype was tested by a professional beekeeper and approved for its usability. The field assessments confirmed the hive design and the quality of encoding and sampling. Automatic analysis of honeybee dances is divided into two main steps. The first step is the automation of recording and the second is the automation of decoding. Here in the current article, we have covered the first step and provided the necessary materials for the second. To go further the constant video quality and exposition make it possible to process the video with automated detection software to improve the monitoring efficiency and to create large data sets to train artificial intelligence models to decode the dances and monitor colony activity. The underlying objective is to provide a revolutionary tool to automate the first “send” phase of the behavioral chain to help searchers to improve knowledge on the second “search” phase and third “attract” phases by producing search area maps to reduce the scope of their research in the field. This tool should ultimately lead to a detailed analysis of the melliferous resources present in a territory. This prior knowledge is essential for all agroecological developments aimed at improving melliferous potential, in particular through the practice of the ApiNutriCulture.

## Figures and Tables

**Figure A1:**
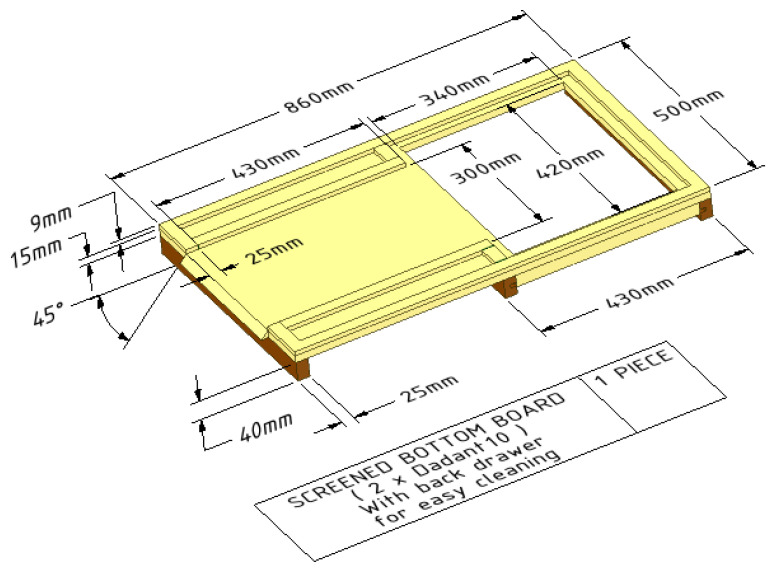
Screened bottom board with back drawer for easy cleaning.

**Figure A2:**
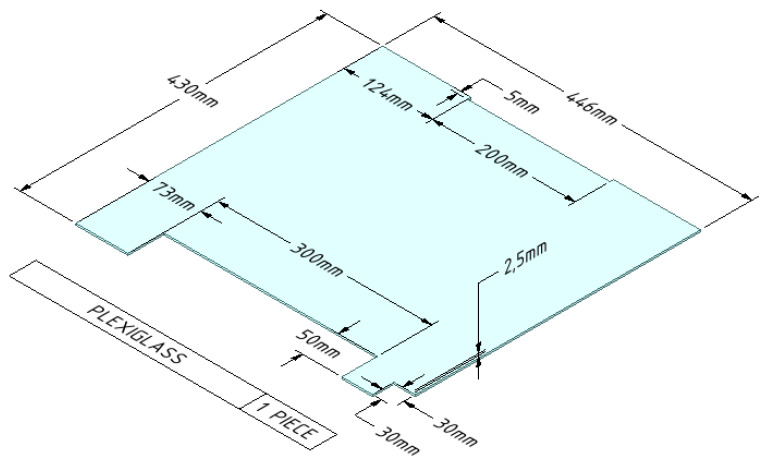
Plexiglass.

**Figure A3:**
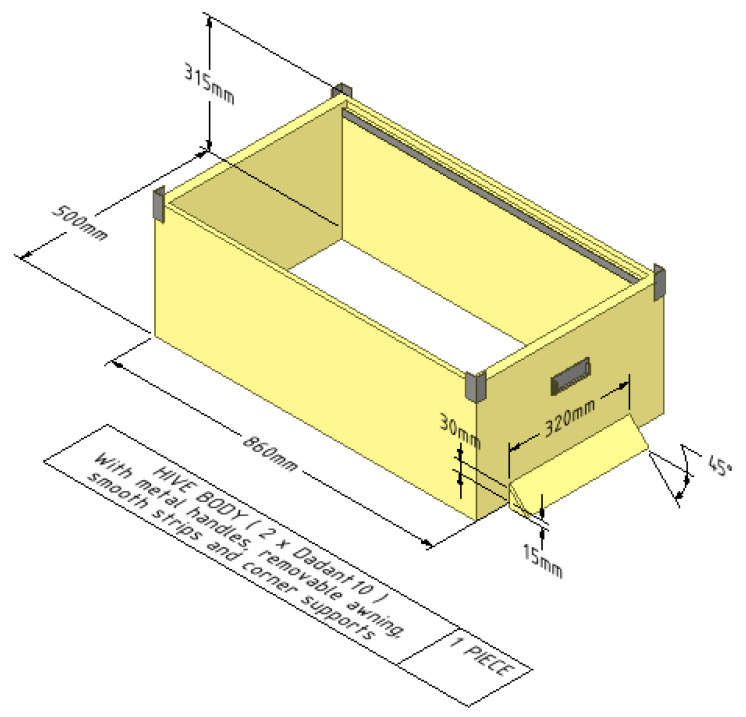
Hive body with metal handles, removable awning, smooth strips and corner supports.

**Figure A4:**
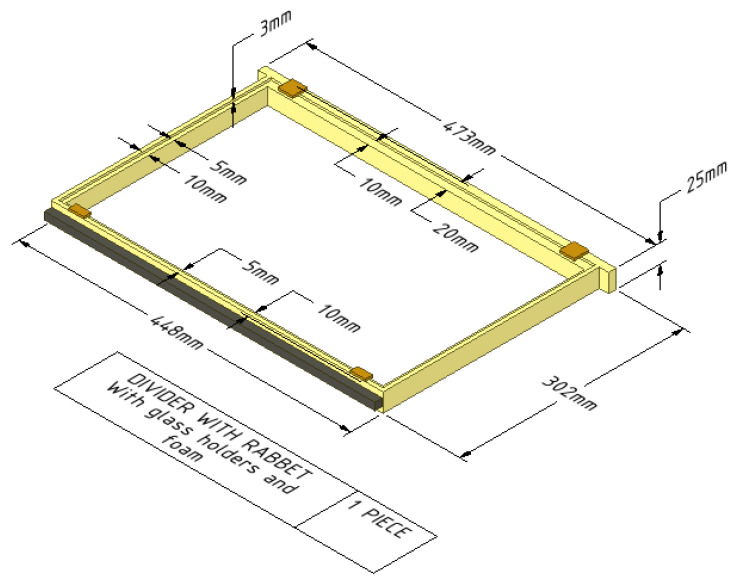
Divider with glass holders and foam.

**Figure A5:**
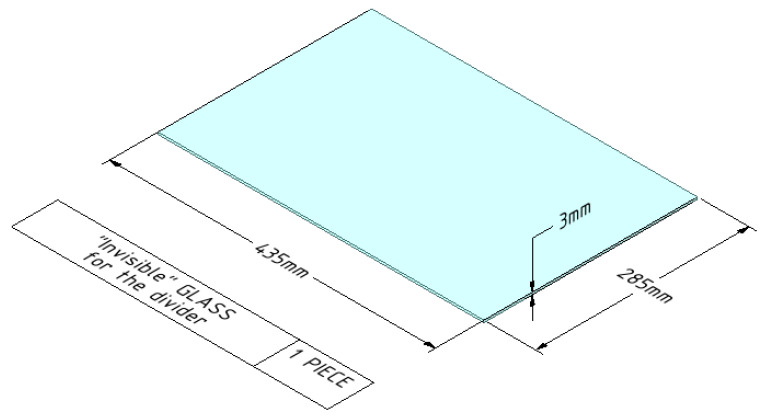
“Invisible” glass.

**Figure A6:**
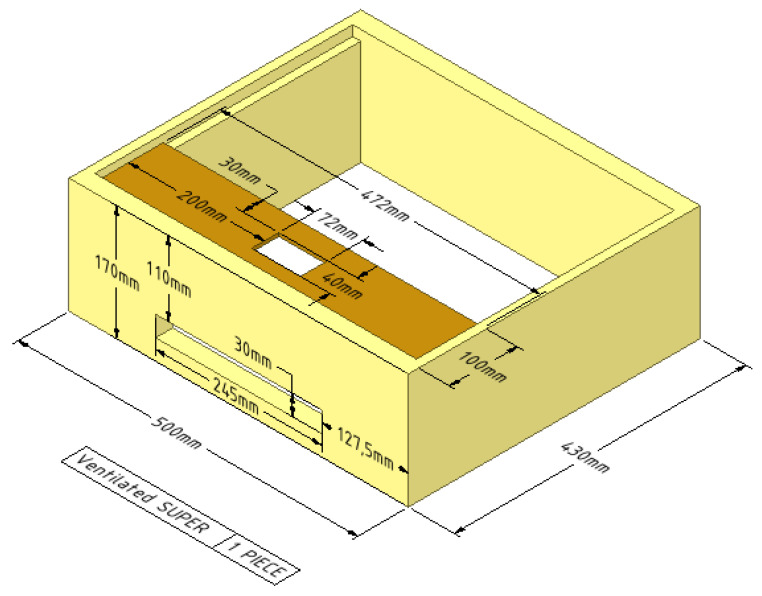
Ventilated super.

**Figure A7:**
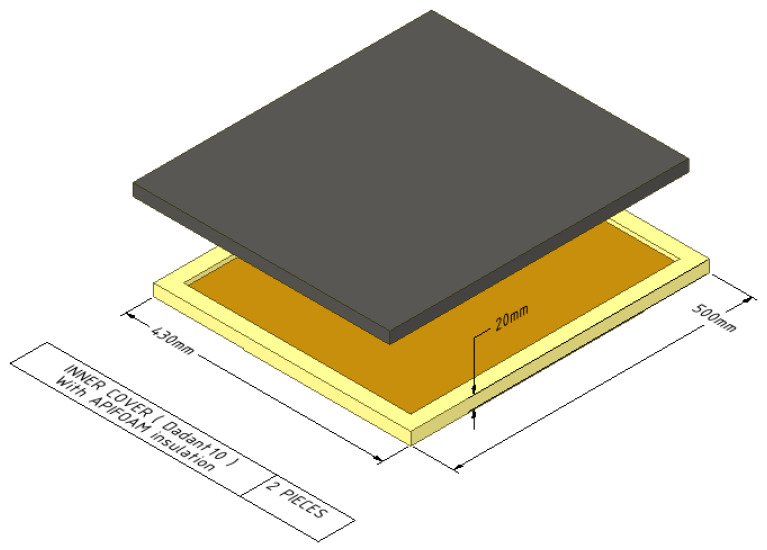
Inner cover with insulation.

**Figure A8:**
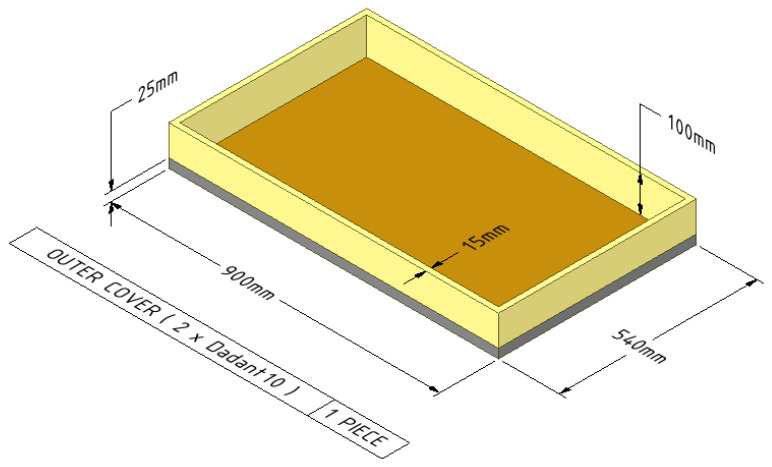
Outer cover.

## Data Availability

https://doi.org/10.5281/zenodo.7415701.

## Abbreviations

The following abbreviations are used in this manuscript:

ApiNutriCulture	Cultivation of Nutrients (plants) for Apis (bees)
CSI	Camera Serial Interface
FPC	Flexible Flat Cable
GPIO	General Purpose Input/Output
HFOV	Horizontal Field Of View
INRAE	French National Research Institute for Agriculture, Food and the Environment
IR	InfraRed
MCU	MicroController Unit
NAS	Network Area Storage
NOIR	NO InfraRed
PWM	Pulse Width Modulation
LED	Light Emitting Diode
RPI	Raspberry PI

## Appendix A. GeoDanceHive Dimensions

The light brown wooden parts are in Weymouth pine (Pinus strobus) to gain lightness. The brown wooden parts are made of plywood. The glass holders are made in MDF (Medium Density Fiberboard). The dark brown wooden parts are made of oak for better resistance. The weight of an empty GeoDanceHive (without frames) is around 17 kg against 11 kg for a simple hive made of the same materials. These plans were made with DesignSpark Mechanical 5.0.
